# The Influence of Supportive and Ethical Work Environments on Work-Related Accidents, Injuries, and Serious Psychological Distress among Hospital Nurses

**DOI:** 10.3390/ijerph15020240

**Published:** 2018-01-31

**Authors:** Maki Tei-Tominaga, Miharu Nakanishi

**Affiliations:** 1 Faculty of Nursing, Kyoto Tachibana University, Kyoto 607-8175, Japan; 2 Mental Health and Nursing Research Team, Tokyo Metropolitan Institute of Medical Science, Tokyo 156-8506, Japan; nakanishi-mh@igakuken.or.jp

**Keywords:** nurse, work-related accident or injury, psychological distress, occupational health and safety, social capital, ethical climate, work environment

## Abstract

The healthcare industry in Japan has experienced many cases of work-related injuries, accidents, and workers’ compensation claims because of mental illness. This study examined the influence of supportive and ethical work environments on work-related accidents, injuries, and serious psychological distress among hospital nurses. Self-reported questionnaires were distributed to nurses (*n* = 1114) from 11 hospitals. Valid responses (*n* = 822, 93% women, mean age = 38.49 ± 10.09 years) were used for analyses. The questionnaire included items addressing basic attributes, work and organizational characteristics, social capital and ethical climate at the workplace, psychological distress, and experience of work-related accidents or injuries in the last half year. The final model of a multivariate logistic regression analysis revealed that those who work less than 4 h of overtime per week (OR = 0.313), those who work on days off more than once per month (OR = 0.424), and an exclusive workplace climate (OR = 1.314) were significantly associated with work-related accidents or injuries. Additionally, an exclusive workplace climate (OR = 1.696) elevated the risk of serious psychological distress. To prevent work-related compensation cases, which are caused by these variables, strengthening hospitals’ occupational health and safety is necessary.

## 1. Introduction

Compared to other industries, the healthcare industry has significantly higher rates of work-related accidents, injuries, and illness in many countries [1,2,3]. In the U.S., hospitals reported 6.8 injuries and diseases per 100 workers, whereas in the manufacturing industry and construction sector, the rates were 4.3 and 3.9 per 100 workers, respectively, in 2011 [2]. Among all work-related accidents and injury cases in 2016 in Japan (*n* = 7361) for which workers needed more than 4 days absence from work, the highest number of cases (*n* = 1718) was in the healthcare and hygiene industry, whereas there were 1425 cases in the manufacturing industry and 622 cases in the construction sector [3]. Musculoskeletal disorders such as back pain account for more than 60% of injury cases and this trend remained unchanged in the last decade [4,5]. Considering that the work-related injury rate is a major reason nurses leave the profession [6], it is critically important to intervene in issues of occupational health and safety within the healthcare industry in Japan, a sector that has experienced a chronic shortage of nurses [7].

Moreover, in Japan, work-related mental illness has become an additional issue for workers in the healthcare and welfare industry, since compensation cases due to mental illness (e.g., depression, etc.) have been increasing. The healthcare and welfare industry has been identified as the highest ranking in terms of the number of workers’ compensation cases for more than five years [8,9]. “Trouble with a supervisor” and “harsh harassment, bullying, and violence” were ranked first and second as related events, respectively, in 2016. In addition, “nurse” ranked seventh among all occupations [8]. From these data, it can be inferred that work-related mental illness cases among hospital nurses may be related to social-relational aspects of work such as ethical behaviour at the workplace.

In hospitals, workers are at risk for the following potential types of hazards: biological (e.g., needle stick injuries), ergonomic (e.g., patient handling), chemical (e.g., chemicals used for disinfecting and sterilizing), physical (e.g., radiation from X-rays and radioisotopes), psychosocial (e.g., stress), and mechanical (e.g., slips, trips, and falls) [10,11]. Regarding work-related injuries in hospitals, McCaughey et al. (2016) [12] identified four antecedents of injury among healthcare employees: individual characteristics and three categories using a framework of the National Institute for Occupational Safety and Health (i.e., organization of work such as shift work, job characteristics such as physical and psychological demands and social-relational aspects of work, and safety programs and training). Additionally, a previous systematic literature review revealed that hospital shift work has been associated with a higher risk of work-related injuries among nurses [13].

In their review study [12], McCaughey et al. also showed that having peer support [14] and collaboration among colleagues [15] decreased the risk of injury among nurses, and that nurses’ aides with higher informal social status had greater access to help from co-workers and reduced exposure to injury risk [14]. The authors inferred from these findings that social-relational aspects of work such as informal social status, a supportive culture, and social support may have an effect on injury risk among employees [12]. Considering issues of work-related accidents, injuries, and mental illness in the healthcare industry in Japan, it is essential to foster supportive and ethical work environments. Furthermore, it is necessary to reveal the influence of social-relational aspects of work among hospital nurses.

In this context, the concept of “social capital”, which consists of mutual understanding, shared aims, and unifying members of social networks and communities [16,17], is beneficial in terms of social-relational aspects of work. Previous studies revealed that social capital in the workplace at hospitals was related to risk factors of psychological health status among workers including healthcare professionals [18,19,20,21]. Specifically, the odds for new physician-diagnosed depression and antidepressant treatment were 30–50% higher for public sector employees with low vertical or horizontal workplace social capital than for their counterparts with high social capital at the workplace [21]. Furthermore, social capital is negatively associated with emotional exhaustion among nurses [19]. 

On the other hand, researchers identified that the mechanisms of social capital could also have other less desirable consequences such as exclusion of outsiders [22,23]. In contrast to a supportive work environment, social exclusion, which has been identified as a negative consequence of social capital, may have adverse psychological and behavioural effects on nurses in hospitals. A previous review study [24] indicated that social exclusion thwarts the need to belong, and being ignored, excluded, and/or rejected signals a threat that promotes pain and distress for adaptive survival. While employees have a stronger intention to quit their jobs when they experience exclusion at the workplace [25], these ethical issues also showed significant relationships with intention to leave one’s current position among nurses and social workers [26]. Furthermore, previous studies revealed notable adverse effects on the brain, such that social exclusion echoed the effects of physical pain [27], and had a worse impact on victims’ self-esteem than did bullying [28].

Thus, we hypothesized that supportive (e.g., high social capital at the workplace) and unethical (e.g., social exclusion at the workplace) work environments, which reflect social-relational aspects of work [12], would be associated with experience of work-related injuries, accidents, and serious psychological distress (SPD) among hospital nurses, which, in turn, could become workers’ compensation cases. Few studies have examined these relationships simultaneously; therefore, our findings provide meaningful insight into the field of occupational health and safety in Japan, which has experienced noteworthy staff shortages due to work-related injuries, accidents, and mental illnesses.

## 2. Materials and Methods

### 2.1. Design, Participants, and Settings

We conducted quantitative research using a cross-sectional survey with convenience sampling. With cooperation from a nursing association in a prefecture in Japan, eleven hospitals agreed to participate. The participants were all nurses from these hospitals.

### 2.2. Data Collection

From September to November 2015, self-administered questionnaires (to be returned anonymously) were distributed to all nurses from 11 hospitals (*n* = 1114). If the nurses agreed to participate, they completed the questionnaire and returned it using a sealed envelope. Among these, 917 participants returned their questionnaires (response rate = 83%); however, only questionnaires with complete data (*n* = 822) were used for analyses (valid response rate = 74%).

### 2.3. Measures

The anonymous self-administered questionnaire written in Japanese included items addressing demographic variables (e.g., age, sex, marital status, education, job position, length of experience at one’s current position, and organizational tenure) and organizational characteristics (e.g., organizational type and hospital size) as control variables. In addition, we asked about participants’ average overtime worked per week and frequency of working on one’s days off per month, and used an original scale to assess supportive environment in the workplace, to assess the independent variables.

For the dependent variable, we included the question, “Have you experienced a work-related accident or injury in the last half year”? As indicated in a previous study [29], government statistics may be limited [3] with regard to capturing accidents, injuries, and illnesses involving less than three days’ absence from work; while these occur more frequently than those causing longer absences, they are not taken into account. Therefore, we used self-reported data to assess work-related accidents and injuries. We also included a scale assessing the presence of psychological distress for the assessment of SPD.

#### 2.3.1. Social Capital and Ethical Climate at the Workplace of a Hospital (SEW)

To assess supportive and ethical work environments, we used the 20-item SEW that has confirmed high internal reliability, criterion-related validity, and construct validity [30] (see Table A1 in Appendix A). The items, which were developed in reference to previous studies [31,32,33,34], were selected as key elements for nurses’ supportive work environment through a three-round panel survey using the Delphi technique with nursing directors. It consists of three sub-scales: “social capital in the workplace” (nine items; e.g., “Overall, nurses are trustworthy”), “exclusive workplace climate” (five items; e.g., “Those who make an error at the workplace are strongly blamed”), and “ethical leadership” (six items; e.g., “Leaders express their understanding over staff nurses’ rights”). Each item was evaluated using a 7-point Likert scale ranging from 1 (totally disagree) to 7 (totally agree). We used each sub-scale’s sum score to calculate the mean per sub-scale. The higher the mean score of two sub-scales (social capital in the workplace and ethical leadership), the greater the participants’ perceived favourable characteristics, and the higher the mean score of one sub-scale (exclusive workplace climate), the greater the participants’ perceived unfavourable work environment characteristics.

#### 2.3.2. The Presence of SPD

To assess the presence of SPD, we used the K6 scale [35,36,37], which is a psychometrically validated epidemiologic screening measure that is highly correlated with diagnostic measures of major depressive disorder and other mental disorders. Participants were asked whether they had recently experienced symptoms or behaviour related to depression or anxiety. Each item was evaluated using a 4-point (1–4) Likert response scale (most of the time, some of the time, a little of the time, and never). The sum of the six questions (coded 0–4 points) was used for screening SPD. Higher sum scores indicated greater psychological distress. We used a cut-off score of 13 as those who had experienced SPD [35,38,39].

### 2.4. Ethical Considerations

Approval for this study was obtained from the institutional ethics committee at Tokyo Gakugei University in 2015 (#08015). All hospital nurses in this survey were not required to provide written consent because return of the questionnaire constituted implied consent. Participants were informed about the voluntary nature of participation (e.g., they were free to withdraw at any time), and assured confidentiality in the handling of data.

### 2.5. Data Analysis

First, we calculated descriptive statistics for individual attributes, employment, and organizational characteristics. Next, we calculated descriptive statistics and Cronbach’s alpha coefficients of dependent and independent variables. As a preliminary analysis to identify the primary variables for a multivariate logistic regression analysis, we examined coefficients for the association between the two dependent variables (the experience of work-related accidents or injuries in the last half year and the presence of SPD) and the nominal data of each variable. Additionally, we calculated Spearman’s correlation coefficients between each dependent variable and SEW sub-scales. Finally, we performed a multivariate logistic regression analysis to examine factors related to the two dependent variables.

A previous study revealed that events per variable values of 10 or greater indicate no major problems (e.g., the Wald statistic was conservative under the null hypothesis, the paradoxical associations, etc.) [40]; therefore, all independent variables were entered into the equation using a stepwise method (backward selection method) after controlling for basic attributes and employment characteristic variables, which were selected based on the results of the preliminary analysis. The odds ratio and 95% confidence intervals for each control and independent variable were calculated for dependent variables. All statistical analyses were performed with IBM SPSS 25.0 (IBM Japan Inc., Tokyo, Japan). A *p*-value < 0.05 was regarded as significant.

## 3. Results

### 3.1. Participants’ Characteristics

Participants’ basic attributes are shown in Table 1. It should be noted that 5% (*n* = 42) of the participants answered that they had experienced work-related accidents or injuries in the last half year. Thirty-four percent worked overtime more than 5 h per week, and 28% worked on days off more than once per month.

### 3.2. Descriptive Statistics and Reliability of Each Scale and the Association between Dependent Variables

The descriptive statistics and internal consistency of each scale are shown in Table 2. Additionally, associations between dependent variables are shown in Table 3. The presence of SPD, which occurred in 6% of participants, had significant associations with marital status, hospital type and size, and working on one’s days off more than once per month. The experience of work-related accidents or injuries showed a significant association with participants’ average overtime worked per week.

### 3.3. Correlation Coefficients between Dependent Variables and SEW Subscales

The results of Spearman’s correlation coefficients showed significance between the presence of SPD and all three SEW sub-scales; however, experience of work-related accidents or injuries was only significantly correlated with an exclusive workplace climate (Table 4).

### 3.4. Results of the Multivariate Logistic Regression Analysis

Results of the final model of the multivariate logistic regression analysis are shown in Table 5. Working overtime less than 4 h per week, working on days off more than once per month, and an exclusive workplace climate were significantly associated with experience of work-related accidents or injuries in the last half year. Furthermore, the results after controlling for participants’ basic attributes and employment characteristics are shown in Table 6. Specifically, an exclusive workplace climate elevated participants’ risk for SPD.

## 4. Discussion

Our findings, which revealed that an exclusive workplace climate is a risk factor for both the experience of work-related accidents or injuries and SPD, underline the importance of promoting supportive and ethical work environments for hospital nurses. Regarding work-related accidents, injuries, and illness in hospitals, four antecedents of injury among healthcare employees (i.e., individual characteristics, organization of work, job characteristics, and safety programs and training) have been identified [12]. In addition, shift work [13] and work pressure and lack of time [41] have been reported as risks of work-related injuries among nurses. Our finding that a social-relational aspect of work (i.e., an exclusive workplace climate) was a risk factor for nurses’ experience of work-related accidents or injuries adds new insight regarding occupational health and safety of hospitals.

While social capital, such as mutual understanding, shared aims, and unifying of social networks and communities [16,17], shows desirable consequences for workers including nurses [18,19,20,21], it can also have less desirable consequences such as exclusion of outsiders [22,23]. Our finding that an exclusive workplace climate was a risk factor for nurses’ experience of work-related accidents or injuries suggests that social exclusion can have adverse effects on occupational health and safety among hospital nurses.

Ethical issues such as an exclusive workplace climate may ultimately influence nursing shortages and indirectly influence the quality of patient care. Previous studies revealed that employees had a stronger intention to quit their jobs when they experienced exclusion at the workplace [25]. On the other hand, a positive and ethical workplace climate for nurses and social workers protects against their intentions to leave [26]. In addition, nurses who experienced workplace ostracism (being excluded and ignored by others [24]), demonstrated an elevated level of silence regarding patient safety [42]. As shown in a previous review, hesitancy to speak up can be a key contributing factor to communication errors [43]. Additionally, while workplace injuries were related to turnover intention [44] and high turnover rates among nurses and nursing assistants [45], the injury rates were also linked to negative patient outcomes [6]. Therefore, an exclusive workplace climate may also indirectly influence patient safety and nurse turnover. 

Moreover, our finding that an exclusive workplace climate was a risk factor for SPD underlines the negative rather than positive effect of social capital on hospital nurses. This supported previous research indicating that social capital can have less desirable consequences such as exclusion of outsiders [22,23]. Thus, the findings in this study confirm the critical influence of social exclusion, which thwarts the need to belong and promotes pain and distress for adaptive survival [24], on nurses’ psychological health. This influence of social exclusion may also result due to nurses working in medical teams. A previous study revealed that those who were excluded showed elevated levels of a biomarker (i.e., salivary cortisol), which increased physiological arousal and stress reactivity related to negative emotion, following a period of exclusion [46]. Additionally, a previous study using functional magnetic resonance imaging revealed that the regions of the brain activated by social pain due to social exclusion are similar to those of physical pain [27], and social exclusion had a worse impact on victims’ self-esteem than did bullying [28].

Taken together, these findings suggest that hospital employers and managers in the healthcare industry, which has experienced a chronic shortage of nurses [7] and faced cases of work-related injury [3,5] and compensation cases due to mental illness in Japan [8,9], need to foster a supportive and ethical work environment. They should also consider influences on hospital nurses’ occupational health and safety, nursing shortages, and the quality of patient care.

In this study, the finding that less than four hours of overtime per week reduced the risk of work-related accidents or injuries highlights the importance of managing nurses’ overtime work. A secondary analysis using data from 340 hospital nurses revealed that among nurses who reported “excessive work”, the average number of hours worked per week beyond those scheduled was more than 4 h, and was related to increased level of acute fatigue [47]. As the Japanese Nursing Association [48] reported, nurses’ subjective fatigue symptoms are correlated with overtime hours, and the more fatigue symptoms nurses have, the more anxious they are about medical accidents. Given these findings, our findings might have been influenced by nurses having less fatigue due to less overwork. 

On the other hand, the finding that the frequency of working on one’s days off per month reduced nurses’ risk contradicts past findings [49]. This might be because nurses do not feel as much pressure when they work a day that deviates from their typical routine. However, there might be the influence of the type of employment such as regular nurses who work less hours than general regular nurses [50]. Furthermore, other factors may have an influence such as type of department [41], type and frequency of work shift [41], and antecedents of injury among healthcare employees [12], as well as differences according to type of hazard [11] and injuries [2,4,5]; thus, further studies are required.

This study had several limitations. First, the participants were all nurses in 11 hospitals in Japan recruited through a convenience sampling method; therefore, the results may be biased and not generalizable to other populations. Second, the use of cross-sectional data does not allow us to draw causal inferences. Additionally, we examined social-relational aspects of work but did not include other antecedents of injury among healthcare employees [12] and factors specific to hospital nurses [13,41]. Further research using a longitudinal design that examines the influence of factors of supportive and ethical work environments and the above-mentioned variables on hospital nurses’ work-related injuries, accidents, and SPD are required. Third, while this study used self-report to measure work-related accidents and injuries, underreporting of occupational injuries is often noted as an issue among healthcare providers [51]; thus, the accuracy of our data is unclear. Fourth, while the SEW showed high internal reliability, further examination is required to confirm its validity. Lastly, it is desirable to examine individual domestic variables (e.g., work family conflicts, family responsibilities, presence of children) and other unethical behaviours in the work environment (e.g., bullying, harassment, incivility) as they may significantly affect nurses’ work-related injuries, accidents, and SPD.

Notwithstanding these limitations, our findings provide meaningful novel insights for the healthcare industry in Japan, which is burdened with an increase in work-related compensation cases and staff shortages. Further studies are needed to investigate the generalizability of these results.

## 5. Conclusions

We examined the influence of supportive and ethical work environments in terms of social-relational aspects of work on both the experience of work-related accidents or injuries and SPD among hospital nurses in Japan. Results of a multivariate logistic regression analysis showed that those who worked overtime less than 4 h per week, those who worked on their days off more than once per month, and an exclusive workplace climate were significantly associated with the experience of work-related accidents or injuries in the last half year. Additionally, an exclusive workplace climate elevated nurses’ risk of SPD. Our findings underline the importance of instilling supportive and ethical work environments to prevent workers’ compensation cases due to work-related accidents, injuries, and SPD among hospital nurses, and added new insight into hospitals’ occupational safety and health.

## Figures and Tables

**Table 1:** Participants’ basic characteristics (*n* = 822).

Variable	Category	*n*	%
1. Sex	Female	768	93
	Male	54	7
2. Marital status	Married	463	56
	Single	359	44
3. Education	Junior college or vocational school equivalency degree	710	87
	College graduate or higher	50	6
	Other	62	8
4. Job position	Staff nurse	711	86
	Head nurse or chief nurse	59	7
	Director or vice director of nursing service department	52	6
5. Hospital type	Public hospital	271	33
	General private hospital	490	60
	Other (psychiatric, etc.)	61	7
6. Hospital size	Fewer than 100 beds	239	29
	100 to 199 beds	220	27
	More than 200 beds	363	44
7. Average overtime/week	None	137	17
	1–4 h	402	49
	5–9 h	175	21
	10–14 h	66	8
	15–19 h	11	1
	≥20	31	4
8. Frequency of working on days off per month	None	588	72
	One to two days per month	173	21
	Three to four days per month	39	5
	Five to six days per month	11	1
	More than seven days per month	11	1
9. Experiencing work-related accidents or injuries in the last half year	No	780	95
	Yes	42	5
**Variable**	**Mean**	**SD**	**Median**
10. Age	38.49	10.09	38.00
11. Experience at current position (years)	11.49	8.61	10.00

**Table 2:** Descriptive statistics and reliability of each scale and the association between dependent variables (*n* = 822).

Variable	Mean	SD	Items	Range	Cronbach’s α
K6 **^a^**	4.69	4.67	6	0–24	0.90
Social capital in the workplace **^b,c^**	4.69	0.99	9	1–7	0.92
Ethical leadership **^b,c^**	4.55	1.27	6	1–7	0.95
Exclusive workplace climate **^b,d^**	3.15	1.33	5	1–7	0.87

**^a^** Higher sum scores indicated greater psychological distress; **^b^** Sub-scales of the 20-item scale for the Social capital and Ethical climate at the Workplace of a hospital; **^c^** The higher the mean score, the greater the participants’ perceived favourable characteristics; **^d^** The higher the mean score, the greater the participants’ perceived unfavourable work environment characteristics.

**Table 3:** The association between dependent variables and participants’ basic characteristics (*n* = 822).

Variable	Category	The Experience of Work-Related Accidents or Injuries				The Presence of Serious Psychological diStress (SPD) **^a^**			
		No	Yes	Coefficients of Association	*p*-Value	No	Yes	Coefficient of Association	*p*-Value
		(*n* = 780)	(*n* = 42)			(*n* = 767)	(*n* = 55)		
1. Sex	Female	728	40	−0.017 **^b^**	0.627	720	48	0.067 **^b^**	0.056
	Male	52	2			47	7		
2. Marital status	Married	442	21	0.030 **^b^**	0.396	440	23	0.078 **^b^**	0.025
	Single	338	21			327	32		
3. Education	Other	734	38	0.033 **^b^**	0.338	723	49	0.054 **^b^**	0.121
	College graduate or higher	46	4			44	6		
4. Job position	Staff nurse	672	39	0.043 **^b^**	0.216	660	51	-0.049 **^b^**	0.162
	Managerial position	108	3			107	4		
5. Hospital type	Public hospital	255	16	0.035 **^c^**	0.605	239	32	0.146 **^c^**	*p* < 0.001
	General private hospital	468	22			468	22		
	Other (psychiatric, etc.)	57	4			60	1		
6. Hospital size	Fewer than 99 beds	228	11	0.034 **^c^**	0.614	230	9	0.137 **^c^**	*p* < 0.001
	100 to 199 beds	206	14			193	27		
	More than 200 beds	346	17			344	19		
7. Average overtime/week	0–4 h	521	18	0.119 **^c^**	0.003	510	29	0.079 **^c^**	0.075
	5–9 h	158	17			157	18		
	≥10 h	101	7			100	8		
8. Frequency of working on days off per month	None	554	34	−0.048 **^b^**	0.165	557	31	0.090 **^b^**	0.010
	One or more days per month	226	8			210	24		

**^a^** SPD = Yes if total score ≥ 13 on the K6; **^b^** Phi coefficient; **^c^** Cramer’s measure of association.

**Table 4:** Spearman’s correlation coefficients between dependent variables and SEW subscales (*n* = 822) **^a^**.

Variable	(1)	(2)	(3)	(4)	(5)
(1) Experience of work-related accidents or injuries **^a^**	1.000				
(2) Presence of SPD **^b^**	−0.062	1.000			
(3) Social capital in the workplace **^c^**	−0.015	−0.112 **	1.000		
(4) Ethical leadership **^c^**	0.000	−0.098 **	0.726 ***	1.000	
(5) Exclusive workplace climate **^c^**	0.084 *	0.151 ***	−0.467 ***	−0.415 ***	1.000

**^a^** “1” = the group those who agreed with the question, “Have you experienced work-related accidents or injuries in the last half year?”, “0” = the group those who disagreed with this question; **^b^** “1” = total score ≥ 13 on the K6, “0” = total score < 13 on the K6. **^c^** Sub-scales of the Social capital and Ethical climate at the Workplace of a hospital (SEW). * *p* < 0.05, ** *p* < 0.01, *** *p* < 0.001.

**Table 5:** Multivariate logistic regression model for the experience of work-related accidents or injuries in the last half year (*n* = 822) **^a^**.

Variable	Odds Ratio	95% Confidence Interval			*p*-Value
Work characteristics					
Average overtime (0–4 h per week) **^b^**	0.313	0.162	–	0.605	<0.001
Working on days off (more than once/month) **^c^**	0.424	0.186	–	0.963	0.040
Sub-scales of the Social capital and Ethical climate at the Workplace of a hospital (SEW)					
Exclusive workplace climate **^d^**	1.314	1.033	–	1.673	0.026

**^a^** Hosmer-Lomeshow goodness of fit χ^2^ = 11.009, *df* = 8, *p* = 0.201; **^b^** 1 = those who worked less than four hours of overtime per week, 0 = others; **^c^** 1 = those who worked on days off more than once per month, 0 = others; **^d^** Continuous variable.

**Table 6:** Multivariate logistic regression model for SPD (*n* = 822) **^a^**.

Variable	Odds Ratio	95% Confidence Interval			*p*-Value
Sub-scales of the Social capital and Ethical climate at the Workplace of a hospital (SEW)					
Exclusive workplace climate **^b^**	1.696	1.359	–	2.117	<0.001

**^a^** Hosmer-Lomeshow goodness of fit χ^2^ = 7.817, *df* = 8, *p* = .452; **^b^** Continuous variable.

## Abbreviations

The following abbreviations are used in this manuscript:

SPD	Serious Psychological Distress
SEW	Social capital and Ethical climate at the Workplace

## Appendix A

**Table A1:** The 20-item Social capital and Ethical climate at the Workplace of a hospital (SEW) **^a^** scale.

No.	Item	Factor Loading		
		Social Capital in the Workplace	Exclusive Workplace Climate	Ethical Leadership
(1)	Nurses show a great deal of integrity as professionals to one another	0.842		
(2)	Overall, nurses are trustworthy	0.834		
(3)	Nurses pursue the collective goals of their workplace	0.792		
(4)	Nurses share the hospital vision from the medium-term objectives	0.770		
(5)	There is a common goal among nurses	0.726		
(6)	Nurses can rely on co-workers at the workplace	0.637	−0.141	
(7)	Employees help each other out to get the work done	0.463	−0.296	−0.102
(8)	It is possible to maintain good human relationships at the workplace despite differences of opinions	0.435	−0.213	−0.277
(9)	Even if one asks others for assistance, employees do not put themselves at a disadvantage in the workplace	0.401	−0.287	−0.129
(10)	Those who make an error at the workplace are strongly blamed		0.834	
(11)	Those who have different opinions need to be silent and keep themselves in place		0.830	
(12)	New staff are not readily accepted in the team		0.727	
(13)	Those who disagree with the opinions of those in higher positions of authority become outcasts in the workplace		0.700	
(14)	Some employees just do their work and are reluctant to lend a helping hand in the workplace	−0.112	0.676	
(15)	Leaders express their understanding regarding staff nurses’ rights			−0.970
(16)	Leaders treat every nurse with kindness and consideration			−0.965
(17)	Nurses can trust their supervisors			−0.865
(18)	Leaders encourage staff nurses’ ideas in decision making			−0.845
(19)	Leaders value each nurse as competent	0.119		−0.802
(20)	Each nurse feels trusted, valued, and respected by leaders, colleagues, and others	0.319		−0.556
Eigenvalue		10.79	2.48	1.67
Variance explained (%)		49.06	11.29	7.61
Cumulative variance explained (%)		49.06	60.35	67.96

**^a^** Results of a factor analysis using data from 779 hospital staff nurses. Each item was evaluated using a 7-point Likert scale ranging from 1 (totally disagree) to 7 (totally agree)*.*
